# Neutron tomography and image registration methods to study local physical deformations and attenuation variations in treated archaeological iron nail samples

**DOI:** 10.1007/s00339-024-07990-x

**Published:** 2024-11-02

**Authors:** Mahdieh Shakoorioskooie, Elodie Granget, Ocson Cocen, Jan Hovind, David Mannes, Anders Kaestner, Laura Brambilla

**Affiliations:** 1 PSI Center for Neutron and Muon Sciences, Forschungsstrasse 111, 5232 Villigen, Switzerland; 2 Haute Ecole Arc Conservation-Restauration, HES-SO University of Applied Sciences and Arts Western Switzerland, Espace de L’Europe 11, 2000 Neuchâtel, Switzerland; 3 Tribology and Interfacial Chemistry Group, SCI-STI-SM, Institute of Materials, École Polytechnique Fédérale de Lausanne, Station 12, 1015 Lausanne, Switzerland

**Keywords:** Image registration, Neutron computed tomography, Corrosion, Archaeological iron, Heritage conservation

## Abstract

This study presents a preliminary examination of the effects of environment changes post-excavation on heavily corroded archaeological Roman iron nails using neutron tomography and image registration techniques. Roman nails were exposed to either a high relative humidity environment, or fast thermal drying as primary experiments to show the power of this imaging technique to monitor and quantify the structural changes of corroded metal artifacts. This research employed a series of pre- and post-treatment tomography acquisitions (time-series) complemented by advanced image registration methods. Based on mutual information (MI) metrics, we performed rigid body and affine image registrations to meticulously account for sample repositioning challenges and variations in imaging parameters. Using non-affine local registration results, in a second step, we detected localized expansion and shrinkage in the samples attributable to imposed environmental changes. Specifically, we observed local shrinkage on the nail that was dried, mostly in their Transformed Medium (TM), the outer layer where corrosion products are cementing soil and sand particles. Conversely, the sample subjected to high relative humidity environment exhibited localized expansion, with varying degrees of change across different regions. This work highlights the efficacy of our registration techniques in accommodating manual removal or loss of extraneous material (loosely adhering soil and TM layers around the nails) post-initial tomography, successfully capturing local structural changes with high precision. Using differential analysis on the accurately registered samples we could also detect and volumetrically quantify the variation in moisture and detect changes in active corrosion sites (ACS) in the sample. These preliminary experiments allowed us to advance and optimize the application of a neutron tomography and image registration workflow for future, more advanced experiments such as humidity fluctuations, corrosion removal through micro-blasting, dechlorination and other stabilization treatments.

## Introduction

The preservation and analysis of archaeological artifacts are important in understanding the past. Starting in the Iron Age from ~ 800 until 50 BCE for Central Europe (CE) and throughout antiquity, 50 BCE-400 CE [1], the technological progress brought by high furnaces allowed the widespread use of iron. Because of its strength and availability, it was extensively utilized for various applications in urban engineering, military, or more ordinary household uses [2]. As a result, iron artifacts are commonly unearthed in archaeological sites, offering a tangible connection to past civilizations. Through analyzing artifacts such as Roman iron nails researchers can gain knowledge on ancient manufacturing techniques, usage patterns, and the environmental conditions that these objects have withstood over millennia [3, 4].

The study of corrosion of archaeological artifacts extends beyond merely assessing their condition; it contributes to our understanding of the materials’ long-term behavior under varying environmental conditions. This knowledge is crucial for developing effective conservation strategies that ensure the longevity and integrity of these cultural heritage objects [5]. Moreover, these objects can contribute to predict the future materials’ degradation in various environments, e.g. in civil engineering and nuclear waste management [6].

**Fig. 1:**
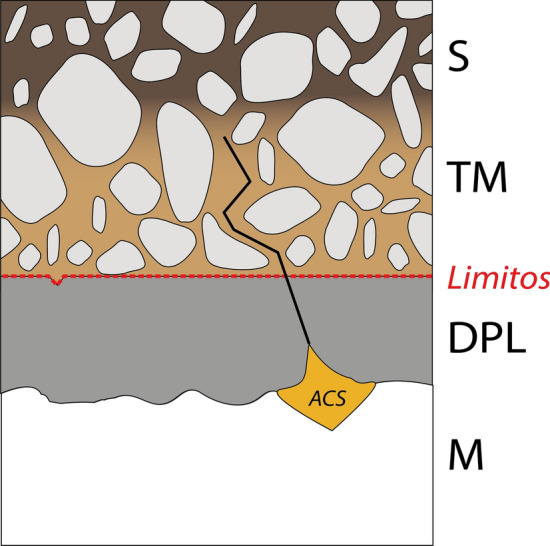
Scheme of a typical corrosion stratigraphy for an archaeological iron object. *S* sediment, *TM* transformed medium, *limitos* original surface of the object, *DPL* dense product layer, *M* remaining metal, *ACS* active corrosion sites at the M/DPL interface

In archaeology, corrosion typically leads to the formation of distinct layers, conventionally labeled as follows [7]: Metal core (M), Dense Product Layer (DPL) often containing active corrosion sites (ACS) at the interface with the metal core, the original object surface (Limitos) containing fabrication and use traces [8], Transformed Medium (TM) above Limitos binding with external markers, and Sediment layer (S) representing the environment (Fig. 1). In conservation laboratories of archaeological institutions, the removal of sediments and TM aim to reveal the Limitos surface. However, the sudden environmental changes that occur during the unearthing of archaeological iron cause significant disturbances to the delicate equilibrium of the objects found in the ground. The high level of moisture contained in artifacts recovered from wet or damp environments, when exposed to the higher oxygen concentration in the air can accelerate corrosion processes, leading to the formation of various iron corrosion products that can further compromise the material’s integrity [9, 10]. Prolonged high relative humidity (RH) levels can lead to the growth of corrosion products such as akaganeite (β-FeOOH) [4, 11]. This volume expansion can exert internal stresses within the artifact, threatening its structural integrity and complicating its preservation [12].

To stabilize archaeological objects post-excavation, intervention on one or more of the three factors causing corrosion, namely relative humidity, oxygen and reactive material, is required. This is achieved by keeping the object in a dry environment (< 10% RH), storing unstable objects in anoxic conditions using oxygen scavengers or transforming reactive corrosion products into more stable ones through desalination and stabilization treatments. Best practice for post-excavation handling of objects places moisture management as the first order of priority [13, 14]. During excavation, the unearthed objects are usually placed in open or perforated plastic bags, while on-site, to avoid condensation. The artifacts are fully dried once at the conservation facility, and subsequently stored in closed bags containing humidity control (silica gel) or oxygen scavengers. Moisture management is often prioritized as it is more cost-effective and allows for easier access to the objects. Nevertheless, oxygen scavengers can be preferred for very unstable objects [15]. It is worth noting that such a conservation protocol is often reserved to small objects that have been inventoried, and very little is done for the batches of common iron objects that remain unstudied [14]. If the combination of moisture and oxygen is a well-known threat to archaeological iron, fast dehydration can lead to equally detrimental damage to these objects. Indeed, this can lead to the formation of cracks and splits within the corroded layers, causing severe structural damage and opening more paths for oxygen and moisture [9, 16]. This paper will explore the effects of extreme cases of high moisture content and fast drying.

As already mentioned, untreated iron archaeological artifacts are unstable, and their advanced corrosion state presents significant challenges to their study and preservation. In that regard, neutron tomography, a non-destructive imaging technique, has emerged as a particularly powerful analysis tool in cultural heritage science for its ability to provide detailed insights into the internal structures of corroded artifacts without causing them any damage. Unlike traditional X-ray imaging, neutron tomography offers enhanced penetration capabilities for metallic objects and is highly sensitive to hydrogenous materials. It is ideal for visualizing organic components and corrosion products within metal artifacts [17, 18]. This capability facilitates a comprehensive examination of the artifact’s internal composition, corrosion patterns, and any embedded organic materials or residues, thereby offering a more complete understanding of the artifact’s condition and history.

The application of neutron tomography to the study of archaeological artifacts represents a significant advancement in the ability to analyze and preserve our cultural heritage non-invasively. By leveraging this technology, researchers can, besides assessing the condition and stability of corroded metal artifacts, also gain insights into the mechanisms of corrosion and its effects on the material over time. This approach allows for the development of more informed conservation practices tailored to the specific needs of each artifact based on its material composition and the nature of its corrosion [19].

This paper presents the use of neutron imaging to study archaeological iron corrosion at various time points, during which the conservation environment is drastically changing. Visualizing the microstructure of these samples and capturing the feature alteration requires nuanced image processing techniques, amongst which image registration is paramount. Image registration involves the alignment of datasets into a unified coordinate system, facilitating the direct comparison of images. This step is crucial for tracking changes in artifacts over time. It enables the identification of small changes within the object, such as variations in density or the presence of new corrosion products and the geometrical changes, which are indicative of ongoing degradation processes or the effectiveness of conservation efforts [20].

In the field of image registration, Mutual Information (MI) metrics have emerged as a cornerstone technique, allowing quantifying shared information between images for effective alignment. Its robustness against various acquisition parameters, such as lighting changes or imaging modality, makes it superior for tasks like comparing neutron tomography images taken at different times [21]-[22]. In fact, MI excels where intensity and geometrical changes are expected, unlike traditional correlation-based methods that assume constant intensity patterns [23]. MI’s statistical dependency assessment handles non-linear intensity variations, making it suitable for images with varying contrasts ([24]. Moreover, MI and correlation methods [25] differ fundamentally in their cost functions. While correlation-based methods assess the direct correlation between pixel intensities, MI employs a cost function based on the individual and joint entropy of the images. MI aims to maximize mutual information between images, enhancing statistical dependency of intensity distributions. This sensitivity to shared information enables effective alignment despite non-linear variations or geometric distortions [21, 22].

The study presented in this paper employs neutron tomography and advanced image processing techniques, particularly image registration, to investigate the impact of environmental conditions on archaeological artifacts. After presenting the objects selected and the environmental exposure set up, it mostly focuses on the development and optimization of the multiple registrations process, where global registration aligns images to a common reference frame, while local registration maps refine deformations, providing insights into localized changes in material composition or structure [26, 27]. Finally, the main takeaways from the preliminary tests presented here will be summarized and perspectives for coming tests will be drawn.

## Materials and methods

### Materials

#### Sample selection and preparation

Multiple heavily corroded Roman iron nails were retrieved from the “Bois de Chatel” forest in Avenches, Switzerland. This site is situated on a hill and showed signs of occupation throughout the Iron Age [28]. It was first a fortified place used by the Gauls (Helvetii) and later a quarry exploited by the Romans. The nails were found with a metal detector in the band of land between the quarry itself and the Roman road, where the workers would have built temporary structures that would move along as the stone exploitation progressed. This band of shallow loamy soil is indeed cribbled with nails left behind. They are found at a depth between 3 and 10 cm under the vegetal layer. Once localized by the metal detector, the nails were taken out of the ground keeping a layer of adhering soil around them. They were directly wrapped in an aluminum foil and placed in tarnish-free plastic bags with oxygen absorbers. This would ensure to keep a similar moisture level and prevent access of oxygen. Once returned to the laboratory, the samples were placed in a glovebox with nitrogen atmosphere, where they were taken out of their initial package to measured their length and take pictures, They were then rewrapped in their aluminum foil and individually placed in a cylindrical sealed chamber made out of a 5 mm thick aluminum of 50 mm diameter, designed to fit the imaging setup (presented in the following section). After the first time-point tomograms were acquired, the nails were carefully unwrapped from their aluminum packing and cleaned with a soft brush to remove loose dirt and debris without disturbing the corrosion layers or the artifact’s integrity. They were then exposed to specific environments for the duration of the experiment and finally returned into the nitrogen sealed chamber. The nails were always kept in anoxic condition unless they were undergoing experimental exposure. Thus, unwanted changes in corrosion products were kept at the minimum.

#### Imposed environmental conditions

Two nails, BdC3 and BdC4, were selected to test extreme environmental conditions expected to cause changes in the corrosion products. They simulate conditions that these artifacts might be exposed to, despite best efforts to guarantee proper preservation of archaeological remains:

1. High relative humidity: BdC4 was exposed to a controlled environment with a high RH of approximately 70–75% for 1 week. This condition aimed to mimic the damp conditions that can accelerate corrosion processes. High moisture conditions can be reached when freshly excavated findings are placed in a sealed plastic bag without proper ventilation. With the rise in temperature, humidity evaporating from the object’s pores can accumulate in the closed bag.
2. Thermal Drying: BdC3 was placed in an oven set at 60 °C for 72 h to simulate fast drying conditions that could potentially cause further cracking or splitting in the corrosion layers. This can happen when objects are left to dry in the sun.

These objects were weighed before and after exposure to these environments to monitor intake or loss of moisture and material.

### Methods

#### Neutron tomography and reconstruction

Neutron tomography of the heavily corroded Roman iron nails was carried out both before and after the experiments in different environmental conditions to reveal the intricate internal structures and patterns of local intensity variations in these artifacts. The neutron imaging acquisition was carried out in the second position of the NEUTRA thermal neutron imaging facility at Swiss spallation neutron source (SINQ) at Paul Scherrer Institute (PSI) [29]. Radiographic acquisition was conducted by a CCD camera coupled with a neutron-sensitive 30 µm thick Gadox scintillator, covering a field of view of 70 × 70 mm^2^. 625 projections with a pixel size of 32.327 µm were acquired, over 360°, with an exposure time of 50 s per projection. The imaging sequence included the acquisition of dark-current (DC) and open-beam (OB) radiographs at the outset, which are needed for the image normalization. This normalization is, however, biased due to the contribution from neutrons, which were scattered from the sample and background. Two reference images (with and without the sample) were obtained with a black body grid installed to measure the scattering contribution [30]. This involved the use of 5-mm thick aluminum frame including a 10 × 10 grid of Ø0.5 mm cylindrical inserts (*black bodies*) made of ^10^B_4_C. The arrangement of these black bodies allowed for precise evaluation and correction of scattering effects, ensuring the clarity and accuracy of the neutron radiographs. Tomographic reconstructions, including the BB corrections, were performed using MuhRec software [31] based on parallel beam filtered back projection (FBP) algorithm. During tomographic acquisitions, the samples were preserved under the protective atmosphere of the custom-designed sealed aluminum chamber (described in Sect. 2.1). A mild positive pressure of 0.2–0.4 bar was imposed in the chamber by purging pure nitrogen gas.

#### Image registration and data analysis

**Fig. 2:**
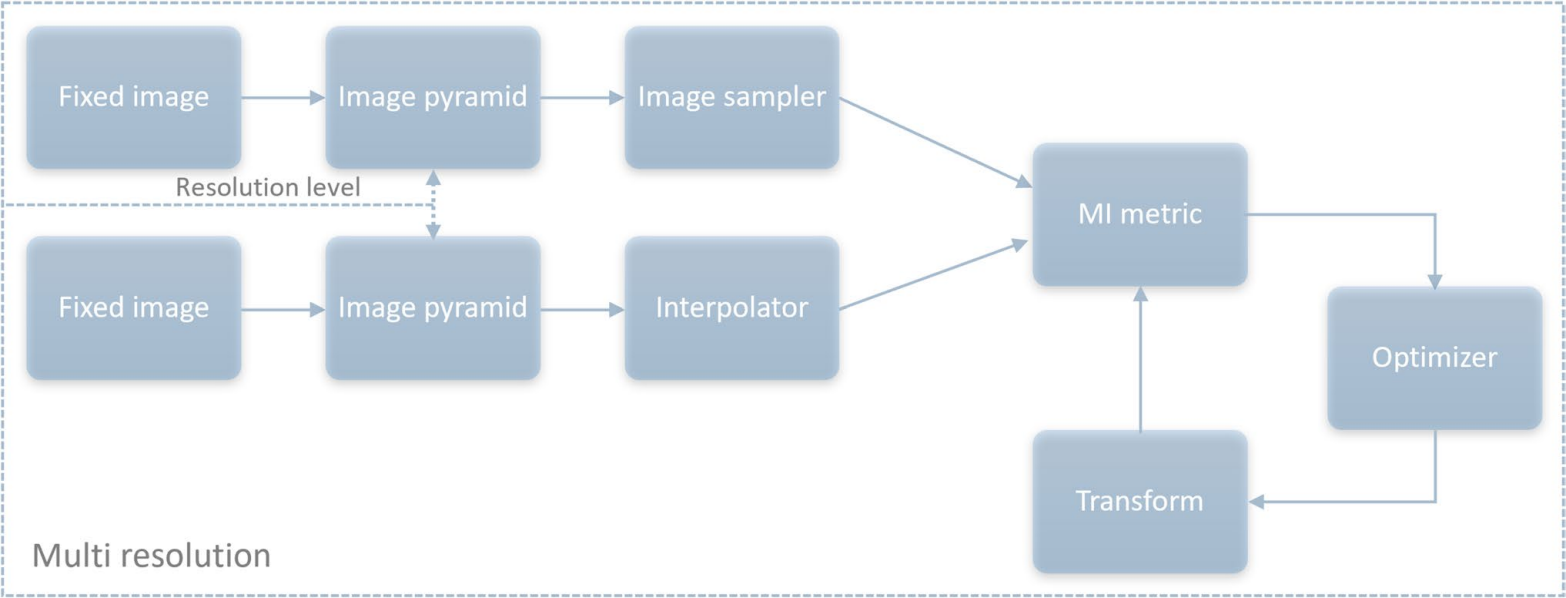
Workflow for 3D image registration as implemented in ElastiX

The 3D image registrations were performed using the Python API of the SimpleElastix image registration library [32]. SimpleElastix extends the Elastix C +  + image registration library, which is based on the Insight Segmentation and Registration Toolkit (ITK) library [33] SimpleElastix integrates Elastix with SimpleITK, a set of bindings to the ITK library available in multiple programming languages [32]. We performed image registration, at three different levels (rigid body, affine and non-affine) for different purposes, based on the workflow schematized in Fig. 2.

##### Rigid body registration

Time-lapse tomography of the same specimen often reveals alignment discrepancies. This implies that a specific reference point on the specimen, such as a corner, might not be in the exact same location across two consecutive tomograms relative to a fixed frame of reference. To address these alignment challenges, rigid body registration was employed to minimize discrepancies between successive tomograms. The initial tomogram was the fixed reference image for each experiment, while the subsequent tomogram was the moving image. The 3D rigid body registration utilized a multi-resolution approach and an image similarity metric grounded in the mutual information (MI) between voxel value populations from the reference and moving tomograms.

##### Affine (global) registration

The next step involved affine transformations, adjusting images for changes in scale, rotation, and shear, providing a more precise alignment that accounts for variations in the imaging setup. The output of the rigid body registration was further registered to the reference tomogram using a global affine transformation (Eq. 1).

The affine transformation, *T*_*Aff*_, was modeled as a combination of rotations, shears, isotropic scaling, and translations, formally expressed as:

1 $$\vec{x}\prime = \vec{T}_{{AFF}} \left( {\vec{x}} \right) = R \cdot G \cdot S \cdot \left( {\vec{x} - \vec{c}} \right) + \vec{t} + \vec{c}$$

where

2 $${\varvec{G}}=\left(\begin{array}{ccc}1& {G}_{12}& {G}_{13}\\ {G}_{21}& 1& {G}_{23}\\ {G}_{31}& {G}_{32}& 1\end{array}\right)$$

3 $${\varvec{S}}=\left(\begin{array}{ccc}{S}_{1}& 0& 0\\ 0& {S}_{2}& 0\\ 0& 0& {S}_{3}\end{array}\right)$$

4 $${\varvec{R}}={R}_{z}(\alpha ){R}_{y}(\beta ){R}_{x}(\gamma )\left(\begin{array}{ccc}\text{cos}\alpha & -\text{sin}\alpha & 0\\ \text{sin}\alpha & \text{cos}\alpha & 0\\ 0& 0& 1\end{array}\right)\left(\begin{array}{ccc}\text{cos}\beta & 0& \text{sin}\beta \\ 0& 1& 0\\ -\text{sin}\beta & 0& \text{cos}\beta \end{array}\right)\left(\begin{array}{ccc}1& 0& 0\\ 0& \text{cos}\gamma & -\text{sin}\gamma \\ 0& \text{sin}\gamma & \text{cos}\gamma \end{array}\right)$$

$G$, $S$ and $R$ are 3 × 3 matrices with elements independent of $x{\prime}$ (i.e., global affine transformation) representing the shear, scaling, and rotation transformations, respectively. Equation 4 defines $R$ as an intrinsic rotation whose Euler angles are $\alpha$, $\beta$, $\gamma$ around the $x, y, z$ axes. The term $\overrightarrow{t}$ in Eq. 1 stands for the translation vector. Since the input to this global affine registration step was a tomogram already rigidly registered against the reference tomogram, the matrix $R$ and the vector $\overrightarrow{t}$ were essentially estimated as the identity matrix and the null vector, respectively, from the affine registration procedure.

##### Non-Affine (local) registration

Finally, to accurately align the tomograms of the samples and to detect and analyze subtle structural changes within the nails, such as local shrinkage or expansion due to the treatments, non-affine local registration was carried out. A rigid body and a global affine transformation lead to displacement vector fields with constant first-order derivatives, which lead to constant strain fields. These transformations are parametrized using B-spline functions (Eq. 5), where control points and coefficients are defined on a lattice overlaid on the fixed image.

B-spline functions includes:

5 $${\overrightarrow{T}}_{\overrightarrow{m}}\left(\overrightarrow{x}\right)=\overrightarrow{x}+{\sum }_{{\overrightarrow{x}}_{k}\epsilon {\mathcal{N}}_{\overrightarrow{x}}}{\overrightarrow{p}}_{k}{\beta }^{3}\left(\frac{\overrightarrow{x}-{\overrightarrow{x}}_{k}}{\sigma }\right)$$

where the ${\overrightarrow{x}}_{k}$ represents the B-spline control points, ${\beta }^{3}\left(x\right)$ shows a cubic multi-dimensional B-spline polynomial, ${\overrightarrow{p}}_{k}$ denotes the B-spline coefficient vectors (the control point displacements), σ is the B-spline control point spacing, and $\mathcal{N}$ the set of all control points within the compact support of the B-spline at $x$. The control points, ${\overrightarrow{x}}_{k}$ are defined on a regular lattice, overlaid on that of the fixed image. The parameters of such a transformation are the ${\overrightarrow{p}}_{k}$. Thus, its total number is triple the number of control points. It can easily fall in the 10^6^ range, indicating that the displacement vector field associated with this transformation may be extremely precise but costly and inefficient. Indeed, iterative approaches are typically used to solve the optimization problem numerically at the core of the registration workflow. This means that a finite difference evolution equation for the parameter vector m is used:

6 $${\overrightarrow{m}}_{k+1}={\overrightarrow{m}}_{k}+{a}_{k}{\overrightarrow{d}}_{k} with k=0, 1, 2, \dots$$

where ${\overrightarrow{d}}_{k}$ indicates the search direction in the parameter vector space at the $k$ -th iteration step and ${a}_{k}$ is a scalar gain factor controlling the step size along the search direction at such step.

However, the large number of parameters involved makes using this B-spline function extremely precise but computationally costly and calls for optimization, such as taking iterative approaches like the stochastic gradient descent (SGD) method (Eq. 7). SGD is preferred for its robustness and requires fewer parameter settings than other optimization methods like quasi-Newton or gradient descent [34]. Therefore, SGD was used for the registration of time series tomograms (Eq. 7).

7 $${\overrightarrow{m}}_{k+1}={\overrightarrow{m}}_{k}+{a}_{k}\overrightarrow{g}({\overrightarrow{m}}_{k})$$

In which the $\overrightarrow{g}\left({\overrightarrow{m}}_{k}\right)=\partial C/\partial \overrightarrow{m}$ evaluated at the current position ${\overrightarrow{m}}_{{k}_{k}}$ in the parameter vector space.

A regularization (or penalty, P) term is introduced to constrain the possible range of $\overrightarrow{m}$ values. The cost function $\mathcal{C}(\overrightarrow{m})$ is thus formulated as:

8 $$\mathcal{C}\left(\overrightarrow{m};{I}_{F},{I}_{M}\right)= -\mathcal{S}\left(\overrightarrow{m};{I}_{F},{I}_{M}\right)+\gamma \mathcal{P}(\overrightarrow{m})$$

where $\gamma$ weighs similarity between the two images against the regularity (often smoothness) of the solution ${T}_{\overrightarrow{m}}(\overrightarrow{x})$. Thus, the registration problem can finally be formulated as the following minimization:

9 $$\hat{m} = {\text{arg}}\;\mathop {{\text{min}}}\limits_{{\vec{m}}} {\mkern 1mu} C(\vec{m};I_{F} ,I_{M} )$$

#### Image distance and similarity metrics

This project uses the mutual information (MI) [35] as the cost function for image registration for all registration steps above. It has been demonstrated that MI performs very well across various applications with single- or multimodal image pairs. MI quantifies the statistical dependence between two images by estimating their joint probability density function (PDF) (Eq. 10). This estimation can be achieved using a Parzen window approach, where B-splines are employed as the smoothing kernel (Eq. 11). In this process, the B-spline Parzen window slides over both the fixed and moving images, facilitating the computation of the joint histogram necessary for MI calculation. With MI as the cost function, the registration task becomes a maximization problem to find the optimal set of transformation parameters. It is sufficient to apply this fine-tuning to a sub-set of the voxels in the fixed image thanks to the registration already performed in the previous steps.

10 $$MI\left(\overrightarrow{m};{I}_{F},{I}_{M}\right)={\sum }_{m\in {L}_{M}}{\sum }_{f\in {L}_{F}}p(f,m;\overrightarrow{m}){\text{log}}_{2}\left(\frac{p(f,m;\overrightarrow{m})}{{p}_{F}(f){p}_{M}(m;\overrightarrow{m)}}\right)$$

By considering $f$ and $m$ two random variables, the images as realizations ${I}_{F},{I}_{M}$, $f$ can be seen as samples from the two respective statistical ensembles and respective PDF, ${p}_{F}\left(f\right)$ and ${p}_{M}\left(m\right)$ and subsequently their joint $p\left(f,m\right)$. MI is calculated based on the PDFs of their voxel values ${L}_{F},{L}_{M}$. To compute the $\frac{p(f,m;\overrightarrow{m})}{{p}_{F}(f){p}_{M}(m;\overrightarrow{m)}}$ the following operations is carried out:

11 $$p\left(f,m;\overrightarrow{m}\right)=\frac{1}{\left|{\Omega }_{F}\right|}{\sum }_{{\overrightarrow{x}}_{i}\epsilon {\Omega }_{F}}{\omega }_{F}\left(\frac{f}{{\sigma }_{F}}-\frac{{I}_{F}\left({\overrightarrow{x}}_{i}\right)}{{\sigma }_{F}}\right)\times {\omega }_{M}\left(\frac{m}{{\sigma }_{M}}-\frac{{I}_{M}\left({T}_{\overrightarrow{m}}({\overrightarrow{x}}_{i})\right)}{{\sigma }_{M}}\right)$$

where ${\omega }_{F}$ and ${\omega }_{M}$ are fixed and moving B-Spline Parzen windows. The scaling constants ${\sigma }_{F}$ and ${\sigma }_{m}$ must equal the voxel value bin defined by ${L}_{F}$ and ${L}_{M}.$ These follow directly from the voxel value ranges of ${I}_{F}(\overrightarrow{x})$ and ${I}_{M}(\overrightarrow{x})$ and user-defined number of histogram bins. ${\Omega }_{F}$ indicates the set of voxel positions of the fixed image. With MI as the cost function, the registration becomes a maximization problem looking for the optimal set of transformation parameters $\overrightarrow{m}$.

#### Image sampler and interpolator

Luckily, this registration process does not need to be applied to all voxels of the fixed image ${\sum }_{{\overrightarrow{x}}_{i}\epsilon {\Omega }_{F}}()$. Thus, a subset of the image’s voxels is sampled at random on the image lattice, using a specific algorithm [36]. Throughout the optimization procedure, the values of ${I}_{M}$ need to be assessed at non-voxel positions ${\overrightarrow{T}}_{\overrightarrow{m}}(\overrightarrow{x})$, for which voxel value interpolation is required. Distinct interpolation techniques (with distinct quality and speed) are typically used, including nearest neighbor methods, linear interpolators and n-th order B-splines, in order of increasing accuracy and computational complexity [37].

#### Hierarchical schemes for complexity reduction

Generally, multi-resolution registration methods can be categorized into two main hierarchical procedures [38]. One procedure is motivated by “transformation complexity” while the other addresses “data complexity”. The hierarchical approaches aim to reduce the mentioned complexities [39]. In other words, to tackle the transformation complexity, the registration can be done in a stepwise manner, starting from rigid body registration, then doing a global affine and finally performing non-affine registration to register very fine local. To overcome data complexity, during each of the registration steps, the processing of the images can be performed in a multi-resolution manner (going from low resolution to high resolution to increase the details gradually). Regarding data complexity, it is commonly recommended to start the registration not with the original images but with versions with a lower degree of complexity, such as those obtained by some kind of smoothing or down-sampling. A set of images with increasing level of resolution and generated from the same original image is known as a “scale space” of such image. Smoothed and down-sampled versions of an image have reduced complexity and (data) size. Such series of images can be thought of as a pyramid. Various types of pyramids (Gaussian and Laplacian pyramids, spline and wavelet pyramids) and scale spaces (e.g., a morphological scale space) exist, amongst which, the Gaussian pyramid is the most popular approach [40]. The tomograms were registered against reference image according to a non-affine transformation model, parametrized by using the multi-dimensional cubic B-spline model.

#### Local displacement vector field (DVF) and local strain proxy (LSP)

The output of the non-affine registration was used to compute 3D fields providing information about the spatial local distribution of climate-induced deformations. Firstly, the displacement vector field (DVF), and its Euclidian norm (scaler field of magnitude of the non-affine deformation) (Eq. 12).

12 $$\parallel \overrightarrow {u} _{{non - AFF}} \parallel \left( {\overrightarrow {x} ,t_{i} } \right) \equiv \parallel \overrightarrow {{x^{\prime}}} \left( {t_{i} } \right) - \overrightarrow {x} \parallel = \parallel \vec{T}_{{non - AFF}} \left( {\overrightarrow {x} ,t_{i} } \right) - \vec{x}\parallel ,\forall i = 1, \ldots .,N_{t}$$

The second scalar field computed, included the determinant of the Jacobian matrix of the transformation function ${\overrightarrow{T}}_{N-AFF}\left(\overrightarrow{x},{t}_{i}\right),$ hereafter referred to as local strain proxy (LSP):

13 $${J}_{{\overrightarrow{T}}_{non-AFF}}\left(\overrightarrow{x},{t}_{i}\right)\equiv \text{det}\left(\frac{\sigma {\overrightarrow{T}}_{non-AFF}}{\sigma \overrightarrow{x}}\right)\left(\overrightarrow{x},{t}_{i}\right), \forall i=1, \dots , {N}_{t}$$

where

14 $$\frac{\partial {\overrightarrow{T}}_{non-AFF}}{{\partial }_{\overrightarrow{x}}}\equiv \left(\begin{array}{ccc}\frac{\partial {T}_{non-AFF.1}}{{\partial }_{{x}_{1}}}& \frac{\partial {T}_{non-AFF.1}}{{\partial }_{{x}_{2}}}& \frac{\partial {T}_{non-AFF.1}}{{\partial }_{{x}_{3}}}\\ \frac{\partial {T}_{non-AFF.2}}{{\partial }_{{x}_{1}}}& \frac{\partial {T}_{non-AFF.2}}{{\partial }_{{x}_{2}}}& \frac{\partial {T}_{non-AFF.2}}{{\partial }_{{x}_{3}}}\\ \frac{\partial {T}_{non-AFF.3}}{{\partial }_{{x}_{1}}}& \frac{\partial {T}_{non-AFF.3}}{{\partial }_{{x}_{2}}}& \frac{\partial {T}_{non-AFF.3}}{{\partial }_{{x}_{3}}}\end{array}\right)$$

is the Jacobian matrix of the transformation function ${\overrightarrow{T}}_{non-AFF}\left(\overrightarrow{x},{t}_{i}\right), {x}_{1}=x, {x}_{2}=y$ and ${x}_{3}=z.$ Additionally,

15 $$d\vec{x}^{\prime} = \frac{{\partial \vec{T}_{{non - AFF}} }}{{\partial _{{\vec{x}}} }} \cdot d\vec{x}$$

such that

16 $$dV_{{\vec{x}^{\prime}}} \left( {\vec{x},t_{i} } \right) = J_{{\vec{T}_{{non - AFF}} }} \left( {\vec{x},t_{i} } \right) \cdot dV_{{\vec{x}}}$$

With $d{V}_{\overrightarrow{x}}$ indicating the volume of an infinitesimal region about the position $\overrightarrow{x}$ at the time zero (before imposed environmental conditions) and $d{V}_{\overrightarrow{x}{\prime}}$ indicating the volume of the corresponding region at time ${t}_{i}$ (after the experiments, in this case) around $\overrightarrow{x}{\prime}$ in the tomographed volume, being the end position after the non-affine mapping of $\overrightarrow{x}$.

Equation 16 shows that the Jacobian determinant of the non-affine transformation gives a spatial map of the ratio between the local volume at ${t}_{0}$ and ${t}_{i}$ for the infinitesimal region (voxel) before and after induced deformations. Values greater than one indicate a local volumetric expansion, and conversely, values less than one indicate a local contraction and a value equal to 1, indicates absent of local volumetric changes [41].

#### Detecting areas of change using inversely registered images

In this study, we performed a time series analysis on the images of the excavated nails using temporal differential analysis. This analysis revealed not only changes in shape but also image intensity, which indicates changes in the material composition. Most simply, this could be changes in water content but most likely also in the formation of corrosion products. The comparison required photometric corrections of the time frames where the metal core was used as an intensity reference since it is considered constant. The histograms of the moving images were adjusted with respect to the average intensity of the metal core, assumed to remain unchanged between timepoints, and thus serving as a consistent reference point for calibration. The metal core is presumed stable and unaffected by environmental conditions over time, thus serving as a reliable reference for normalization. By linearly shifting the histograms of the two images, based on the transformation required locally for a volume of interest (VOI) in the iron, we ensured that the intensity variations observed post-subtraction were solely attributable to changes in the sample’s phases, such as moisture uptake or loss (not to the fluctuations in the imaging).

## Results

### Material loss and gain (local image intensity or material attenuation variations)

**Fig. 3:**
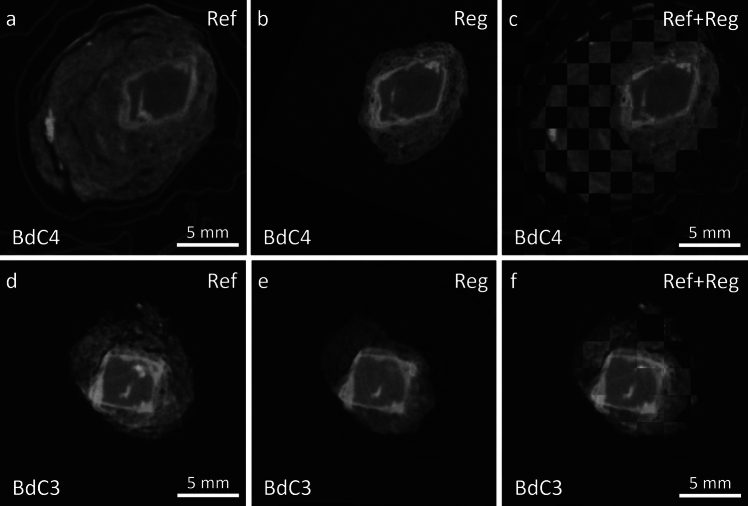
Preliminary registration results, showing the aligned neutron tomogram slices from the Pre-treatment (Ref) and Post-treatment (Reg) states for the BdC4 (first row) and BdC3 samples. The BdC4 sample was exposed to high relative humidity (RH), while the BdC3 sample underwent drying conditions. In this plot, the labels “Ref”, “Reg”, and “Ref + Reg” indicate the reference tomogram, the registered tomogram, and the combined view of both using a checkerboard representation, respectively

Figure 3 presents single selected slices of the post-registration tomograms of both dried and hydrated samples, providing a visual representation of the registration results. Ref images are showing the state of the object pre-exposure, and Reg images, their state post-exposure. The third images in each row show the superposition of both states to provide a better local comparison.

This initial qualitative result of the registration process highlights the effectiveness of our chosen parameters and parameter values, demonstrating that, despite several post-treatment modifications such as the removal of S and partial loss of TM, we have successfully achieved good alignment and registration. Most importantly, the use of Mutual Information (MI) metric has been instrumental in ensuring that the registration is not only accurate but also robust under varying conditions. This outcome validates our approach and confirms the reliability of MI as a metric in complex registration scenarios.

**Fig. 4:**
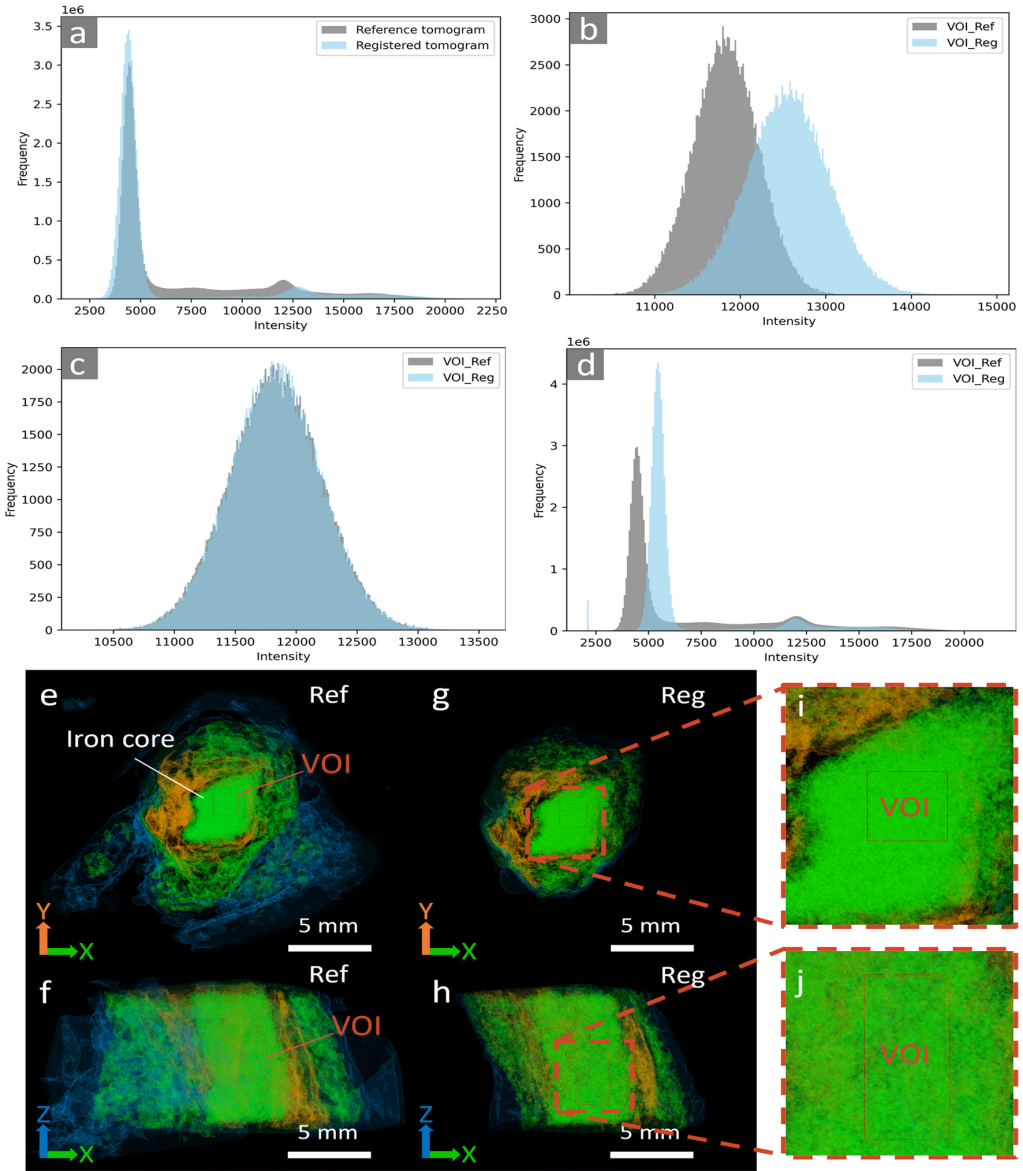
Illustrating the histogram matching process for a small fraction of the BdC4 sample (similar analyses for the full-size BdC3 and BdC4 samples are presented in Fig.10 and Fig.11 in the Supplementary materials): **a** Full histogram of the post-treatment tomogram (Reg) of the BdC4 sample before matching the histograms of the iron core VOIs in both pre-treatment (Ref) and post-treatment stages; **b** superimposed histograms of only the iron VOI before matching; **c** superimposed histograms of only the iron VOIs after matching, showing the transformation of the Reg VOI to match the Ref VOI; **d** full histogram of the Reg tomogram undergoing the same transformation as its VOI; **e**, **f** highlighting the VOI selected in the Ref tomogram and **g**–**j** the corresponding VOI in the exact same region of the sample in the post-treatment (Reg) tomogram

Prior to temporal subtraction, we normalized the histograms of the moving images in relation to the intensity-invariant values from the metal core regions of the samples, as depicted for part of the tomograms of BdC4 in Fig. 4. Similar results for the entire BdC3 and BdC4 samples are in the Appendix A (Fig. 10 and Fig. 11). This figure illustrates the adjustment of histograms, aligning the pixel intensity distributions between the pre- and post-experiment images. By normalizing with respect to the metal core, we effectively remove variations in image intensity that may arise from beam fluctuations during imaging or changes in other imaging setup parameters and artifacts. This allows us to focus more accurately on the genuine local intensity variations that occur due to treatments between the two time points.

In other words, we matched the intensities of the reference and registered images by adapting the histogram of the registered image to the reference one, using the volumes of interest (VOI) selected from the iron core. First the local histogram of the VOI in the registered image is transformed such that it matches the histogram of the same exact VOI in its corresponding reference tomogram (Fig. 4a and b). The successful alignment of the VOI histograms served as a basis for applying the same transformation parameters across the entire tomograms (Fig. 4c and d). By extending the transformation from the VOIs to the entire tomograms, we retained the local contrast in the registered image.

**Fig. 5:**
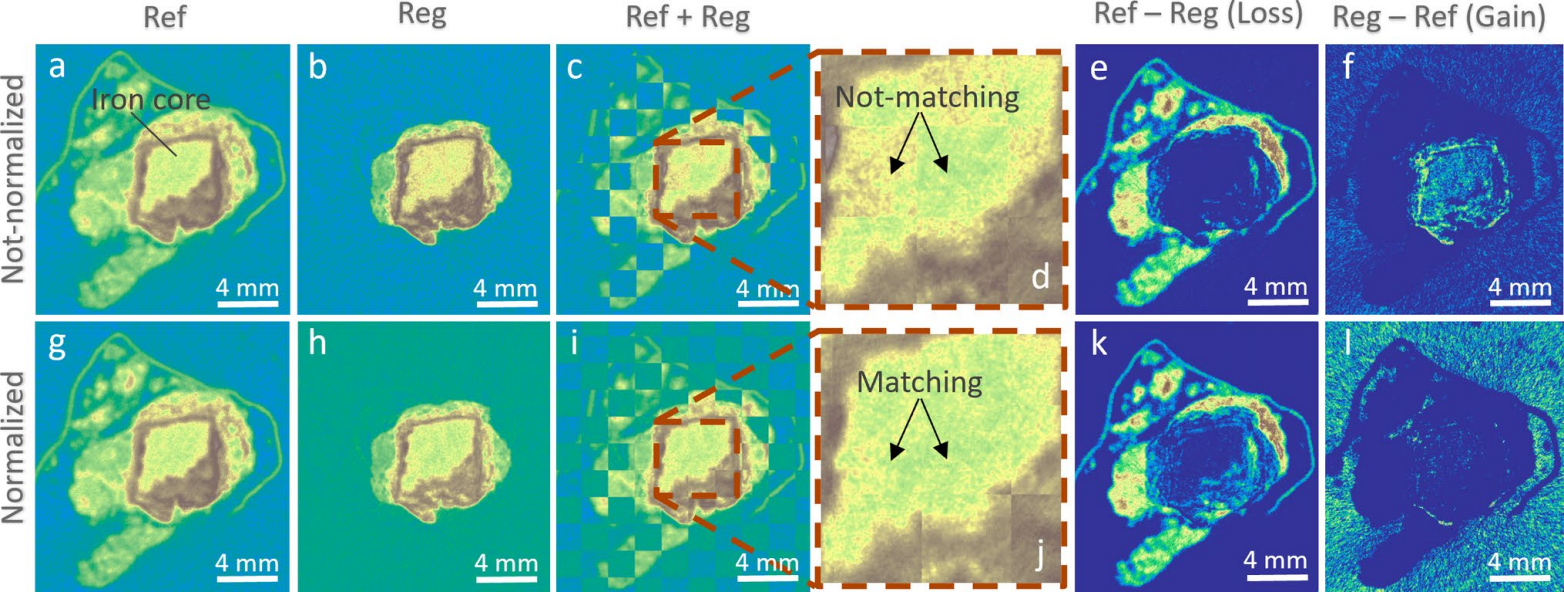
The effectiveness of the histogram matching approach is demonstrated in the modified registered image (Reg) for sample BdC4. **a**-**f** Cross-sectional views before **a**–**c** and after **g**–**i** histogram normalization. **a** and **g** Reference (Ref) image, **b** and **h** Registered (Reg) image, and **c** and **i** Checkerboard overlay of Ref and Reg images, with **d** and **j** zoomed-in regions highlighting non-matching **d** and matching **j** areas. **e** and **k** show the temporal subtraction of Ref—Reg indicating loss, and **f** and **i** show Reg—Ref indicating gain. In other words, the second row illustrates the uniform intensity matching of iron regions post-normalization, in contrast to the initial discrepancies seen in the first row. The improved accuracy in detecting loss and gain post-histogram adjustment confirms the precision of the methodological refinements

The effectiveness of this approach is evident in the modified registered image (only for sample BdC4) in Fig. 5. It is observed in the cross-sectional views that the intensity of iron regions is uniformly matched between the reference and registered images in the second row, in contrast to the initial lack of matching, evident before histogram modifications. This improvement is further highlighted in the checkerboard and its zoomed-in images in the first row, where the discrepancies prior to histogram matching are more apparent, compared to the corresponding modified images in the second row. Additionally, the results of the temporal subtraction, after histogram adjustments, demonstrate that we achieve a more accurate detection of loss and gain in the tomograms as a result of the treatment, confirming the precise impact of our methodological refinements.

**Fig. 6:**
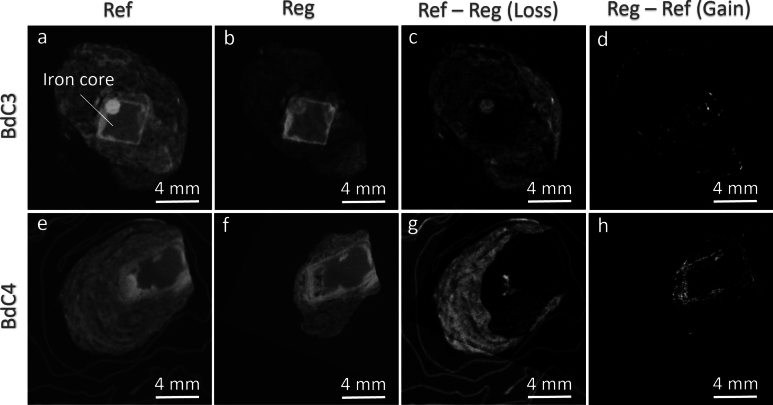
Post-normalization and differential analysis highlighting regions of local attenuation changes indicative of material loss and gain due to treatment in samples BdC3 and BdC4. **a**–**d** Cross-sectional tomogram slices of the dried sample (BdC3): **a** Reference (Ref) image, **b** Registered (Reg) image, **c** Differential image showing loss (Ref—Reg), and **d** Differential image showing gain (Reg—Ref). **e**–**h** Cross-sectional tomogram slices of the high relative humidity (RH) treated sample (BdC4): **e** Ref image, **f** Reg image, **g** Differential image showing loss and **h** Differential image showing gain. For BdC3, areas of decreased intensity in (c) reflect moisture loss in the active corrosion sites (ACS) or in the dense phase layer (DPL) and removal of the sediment layer (S) and transformed medium (TM) during the brushing treatment, while small areas of increased intensity in (d) suggest localized densification, phase transformations or entrapped soil particles. For BdC4, the differential images show significant regions of increased intensity due to moisture absorption by hygroscopic compounds, with heterogeneous intensity variations highlighting changes within the ACS and DPL

After an effective normalization and differential analysis, the resulting images (Fig. 6) highlight regions of local attenuation changes in the tomograms, indicative of material loss and gain due to treatment in both samples. In Fig. 6, we present selected cross-sections (tomogram slices), with each row corresponding to one of our treated samples. For the dried sample (BdC3), the differential images display areas of decreased intensity associated with moisture loss, visually demonstrating the drying effect on the sample’s microstructure. In these loss regions, particularly on the exteriors of the samples, we also observe the intentional removal of the S layer and the accidental loss of poorly adhering parts of the TM. These components were brushed off during the sample preparation for the environment exposure. In the dried sample, we also observe small areas of increased intensity (gain) that could be associated with alterations in density or phase in some material components. In other words, this observation could indicate localized densification or phase transformations that increase the attenuation properties of specific regions, highlighting the complex response of different materials within the sample to the drying process.

Conversely, for the sample exposed to the high RH, the differential images show relatively more areas of increased intensity, indicating further gain from moisture absorption and possible phase alterations at the material level. This gain is attributed to the hygroscopic compounds within the corroded sample, which absorb moisture from the environment, leading to an intensity increase in the tomograms. The heterogeneous nature of these hygroscopic compounds results in a non-uniform intensity variation across the sample, with significant changes occurring inside the sample in the DPL. This seems to be more specifically the case for localized zones of the DPL, close the M interface, hinting at the presence of ACS. These variations are critical in understanding the distribution and extent of moisture absorption, as they will potentially reflect the local strain development (discussed in the next paragraphs and Fig. 8) and phase changes occurring within the iron matrix. To confirm the link between the increased intensity either with the hydration or possible reprecipitation of corrosion products or any phase alterations, ex-situ cross-validation studies are required. This insight into the behavior of the corroded iron under varying humidity conditions is essential for developing preservation strategies and understanding the long-term impacts of environmental exposure on archaeological iron artifacts.

**Fig. 7:**
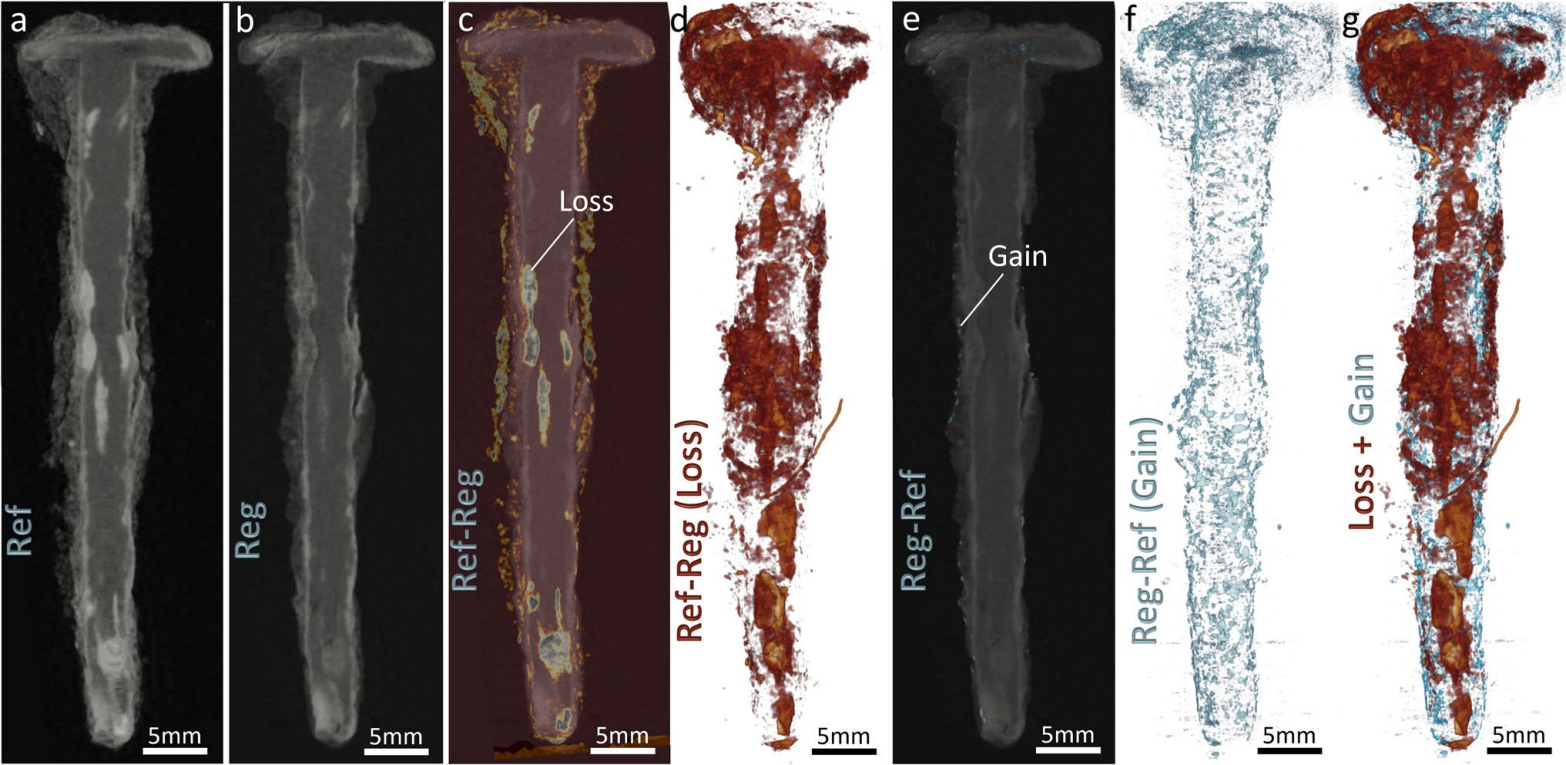
Longitudinal slices and 3D rendered images highlighting zones of material loss and gain in the sample post-treatment. **a** Reference (Ref) image, **b** Registered (Reg) image, **c** Differential image showing loss (Ref—Reg), and **d** 3D rendering of the loss regions, illustrating material removal in the sediment (S) and transformed medium (TM) regions and attenuation reduction due to moisture loss or phase alterations. **e** Differential image showing gain (Reg—Ref), and **f** 3D rendering of the gain regions, indicating minor gains around the sample’s perimeter, potentially due to phase densification or particles entrapped during brushing treatment. **g** Superimposed 3D rendering of both loss and gain regions, providing a comprehensive overview of the sample’s response to treatment

Figure 7c and d highlight the zones that underwent material loss or reduced attenuation, in the longitudinal slices of the samples and corresponding 3D rendered images. The brushed off parts mainly in the exteriors of the sample (S and partially TM regions) and the attenuation reduction due to moisture loss or phase alterations are presented in these figures (as illustrated in the X–Y cross-section of the sample in Fig. 6, first row). In our future studies we will advance our image processing workflow such that we can differentiate these different regions (true alterations due to treatment, from the removed S and TM), using a 3D surface contour encompassing the sample’s volume post-treatment. A rather uniform attenuation reduction (loss) in the TM and DPL region of the sample is observed as opposed to local attenuation reductions observed in the ACS region (Fig. 7c). As mentioned earlier, these losses could be associated to the removal of moisture that likely leads to a densification of the remaining material and potential cracking in the porous regions. In Fig. 7e and f, we can also observe minor gains, rather uniformly observed in the perimeters of the sample. This could be associated to the densification of certain phases, or the particles entrapped in the external layers due to the brushing treatment. Figure 7g illustrates the superimposed 3D rendering of the spatial distributions of both the loss and gain parts in the sample. This visualization provides a comprehensive overview of how different regions within the sample have responded to treatment.

**Fig. 8:**
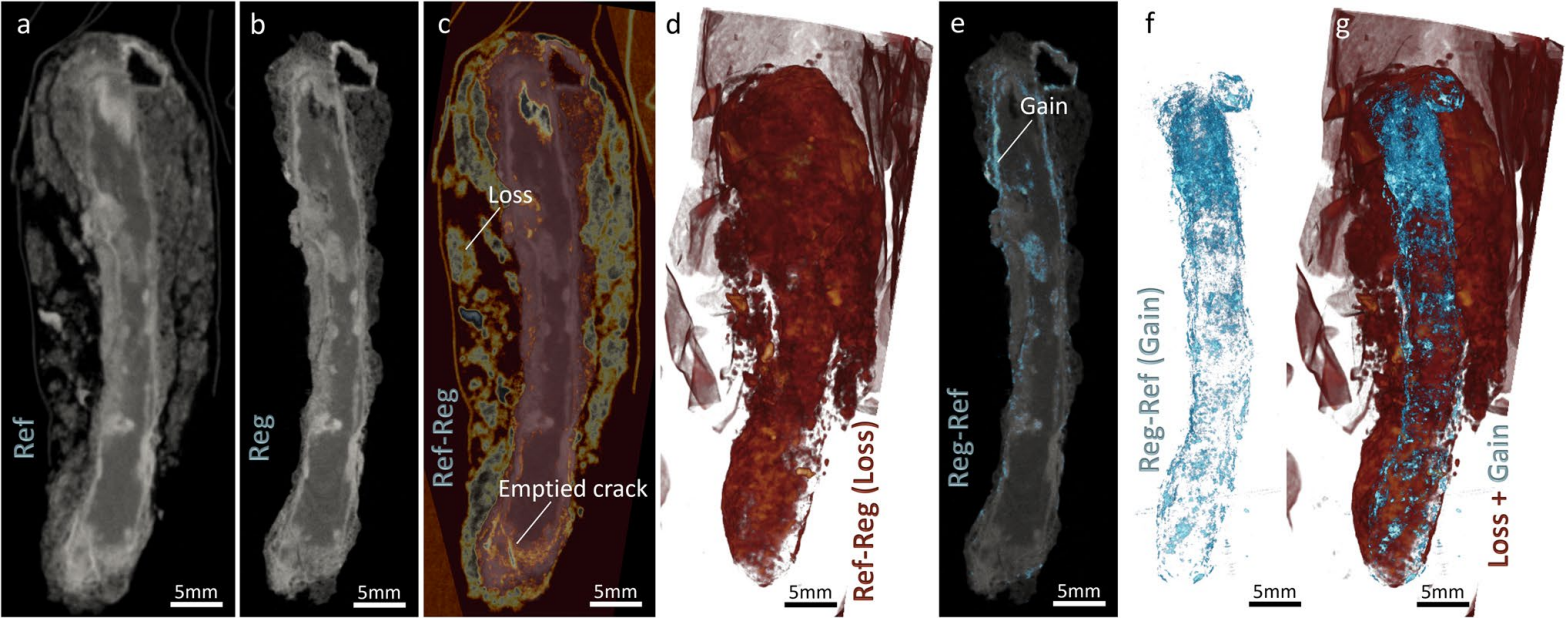
Longitudinal slices and 3D rendered images of the BdC4 sample, which underwent high relative humidity (RH) treatment, highlighting material loss and gain due to moisture absorption and corrosion processes. **a** Reference (Ref) image, showing the exogenous material attached to the sample, including aluminum tape and adhering soil. **b** Registered (Reg) image. **c** Differential image showing loss (Ref—Reg), indicating regions affected by treatment, including the removal of exogenous materials. **d** 3D rendering of the loss regions, illustrating significant attenuation reduction in the active corrosion sites (ACS) present within the remaining metal core (M) and dense product layer (DPL) regions. **e** Differential image showing gain (Reg—Ref), with increased intensity areas due to moisture absorption and rehydration processes. **f** 3D rendering of the gain regions, showing channels of rehydration permeating the iron matrix. **g** Superimposed 3D rendering of both loss and gain regions, providing a comprehensive overview of the sample’s response to high RH treatment.

Figure 8 illustrates similar features for the BdC4 sample, which underwent high RH treatment. For this sample, as mentioned before, the absorbed moisture can lead to further corrosion processes and phase transformations. Furthermore, new corrosion products could also form and occupy existing void spaces in the TM and DPL/ACS. This results in areas of increased intensity in the subtraction images (as seen in Fig. 8e). These areas are clearly observed as channels of rehydration. We can observe how moisture has permeated the iron matrix, potentially leading to localized rehydration and subsequent phase changes.

The BdC4 sample had a significant amount of exogenous material attached to it. Most prominently, a layer of aluminum tape was left on the surface of the foil used to wrap the nail, which is visible on the first tomogram (Fig. 8a). Additionally, there was a lot of soil adhering to the nail (Fig. 8a). These two subtracted materials are highlighted but not differentiable from the losses due to treatment in Fig. 8c and d, where the aluminum tape is visible as a thin layer enveloping the clumps of soil.

Surprisingly, in this sample, we not only observe areas of increased attenuation (gain) but also areas of treatment-affected losses, mostly in the DPL and ACS regions. This could be linked to the re-dissolution of certain corrosion products within the pore space of the sample, however, to truly understand this observation, further complementary ex-situ characterizations will be done in our future study. Nevertheless, this detailed visualization helps in understanding the impact of high humidity on the BdC4 sample, highlighting the intricate interplay between moisture uptake, material degradation, and redistribution.

### Local physical changes

As mentioned in the methods section, following the affine registration, non-affine registration was carried out to capture the local physical changes induced by the treatments or moisture dynamics. By utilizing a higher degree of freedom in the transformation model, non-affine registration can detect subtle local changes in geometry. Figure 7 and Fig. 8 forges the local analysis of the attenuation variations (discussed in the previous section) with the local deformation analysis, namely local displacement vector field (DVF), $\Vert {\overrightarrow{u}}_{non-AFF}\Vert \left(\overrightarrow{x},{t}_{i}\right)$, and local strain proxy (LSP), ${J}_{{\overrightarrow{T}}_{non-AFF}}\left(\overrightarrow{x},{t}_{i}\right),$ both introduced in the methods section. In these images, we obtain a more comprehensive picture of interplay between local gains/losses with the development of local physical changes. Thus, it allows us to better understand how different regions within the sample respond to the environmental changes and helps to identify areas of potential structural weakness or transformation.

**Fig. 9:**
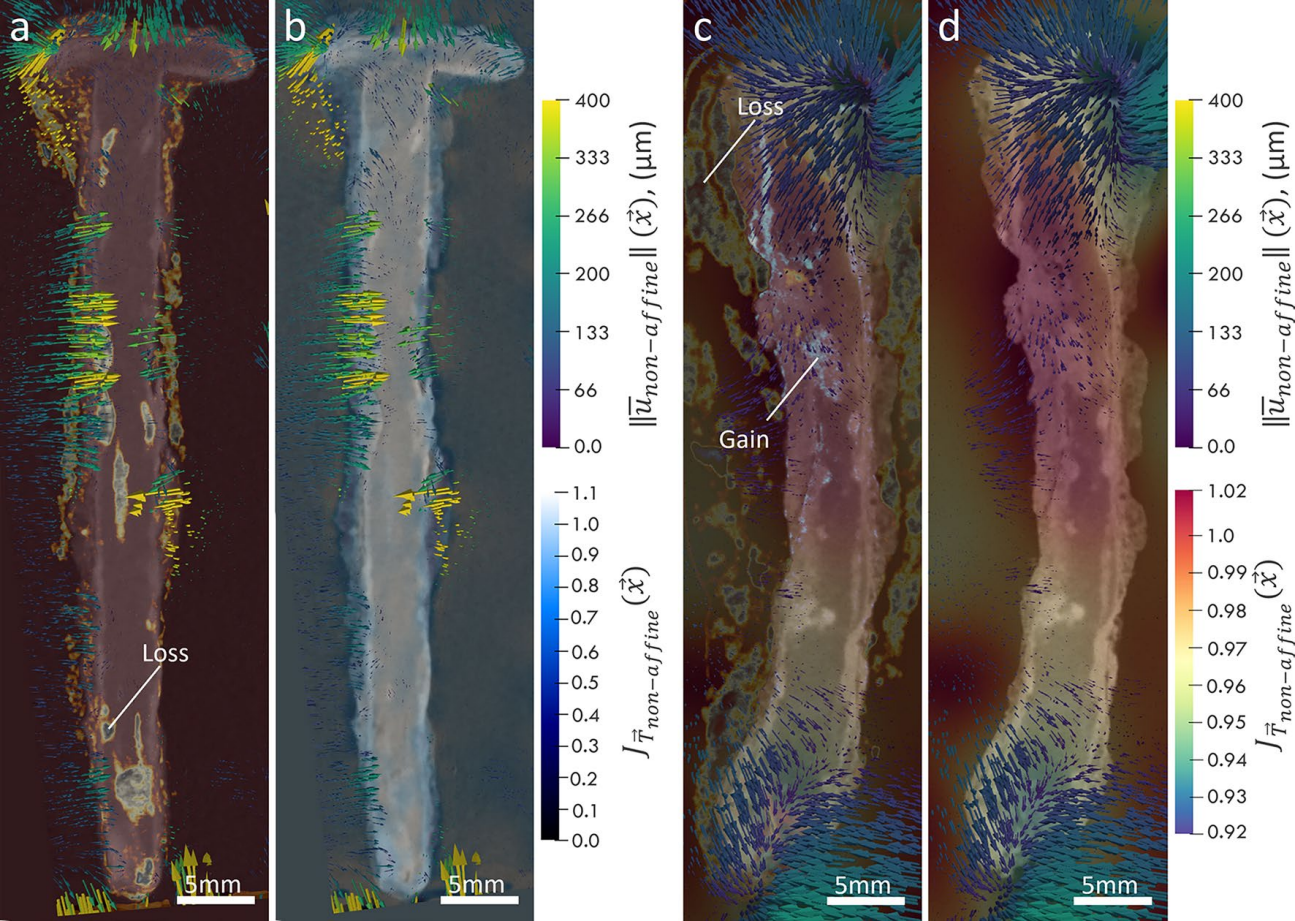
Analysis of local physical changes induced by treatment or moisture dynamics in samples BdC3 and BdC4 using non-affine registration to capture subtle local geometry changes. **a** Superimposed displacement vector field (DVF), $\Vert {\overrightarrow{u}}_{non-AFF}\Vert \left(\overrightarrow{x},{t}_{i}\right)$, over the longitudinal registered (Reg) tomogram slice (also highlighting the loss regions) of BdC3, with inward-pointing vectors in the transformed medium (TM) and dense phase layer (DPL) surrounding the iron core (M). **b** DVF overlaid on the local strain proxy (LSP), ${J}_{{\overrightarrow{T}}_{non-AFF}}\left(\overrightarrow{x},{t}_{i}\right),$, scalar field, showing local shrinkage due to moisture dynamics. **c** and **d** Similar illustrations for the BdC4 sample exposed to high relative humidity (RH). For BdC4, complex spatial distributions of local physical changes indicative of both expansion and contraction, with non-uniform local expansion in the central part associated with considerable moisture uptake by hygroscopic corrosion products

Figure 9a presents the superimposed DVF, in the form of arrows, representing the orientation and magnitude of the local displacement filed, over the longitudinal tomogram slice containing the losses (same as Fig. 7c) for BdC3 sample undergoing drying treatment. Figure 9b, similarly shows the same DVF, however superimposed over the corresponding LSP scaler filed, that quantifies the percentage of local expansion/shrinkage due to moisture dynamics. Distinct color scales are assigned to the DVF and LSP, to ensure clarity in interpreting the overlaid quantities. Similar illustrations are plotted for the BdC4 sample, undergoing high RH exposure, in Fig. 9c and d. Except that in Fig. 9c, in addition to the losses we also demonstrate the gain regions simultaneously.

In the case of the dried sample (BdC3) a predominant shrinkage is observed represented by dominantly inward-pointing vectors and the dark blue coloration in the LSP map, mainly in the DPL region surrounding the iron core (M). These local contractions and their magnitude are well in-line with the local losses taking place in this sample due to the moisture loss and consequent phase transformations. The observation of local shrinkage indicates that the evaporation of moisture from the active corrosion sites (ACS) and the surrounding porous matrix (TM), not only leads to the local attenuation changes but also results in the physical deformations. Weighing this sample indeed indicated a slight weight loss, from 6.237 ± 0.002 g to 6.042 ± 0.002 g, as a result of drying treatment. These products can contract as they lose the bound and adsorbed water within the corrosion layers and any hygroscopic salts present. The rather uniform distribution of the shrinkage areas surrounding the nail suggests that this layer is itself uniform with regards to moisture content. However, at the interface with the remaining metal, localized changes occurred in what could be ACS. These sites are usually composed of hygroscopic and highly reactive products such as akageneite. These products were indeed identified in other nails excavated from Bois-de-Châtel by means of µ-Raman spectroscopy [42].

For the nail sample exposed to the high RH (BdC4), we observed a more complex spatial distributions of the local physical changes. The areas of expansion are mapped as warmer color hue and outward-pointing vectors in Fig. 9c and d. Indeed, the extremities of the nail seem to contract whereas the center of the shaft expands. This kind of behavior is expectable, as in this sample we observe the presence of both gain and losses, associated with both expansion and shrinkage, respectively. The non-uniform local expansion in the central part of this sample, in the layers around the iron core, could be associated to the uptake of moisture through hygroscopic corrosion products, leading to an increase in volume. This suggests a varied distribution of these hygroscopic compounds within the corrosion matrix. More likely, the presence of a highly porous area (crack-like) in the local expansion part in evidence could have facilitated the moisture uptake and concentration in this region. Nevertheless, further similar experiments, along with ex-situ characterizations, will be carried out in our future studies to understand the nature of phases and their chemico-physical behavior.

### Summarizing response of various phases to hydration/dehydration treatments

Following the experiments with imposed environmental conditions—drying and hydration—distinct patterns of change were observed within various phases:

- **Remaining metal core (M)**: As expected, the volume of the intact metal remained largely stable, indicating minimal direct impact from the treatments on the metal’s bulk structure.
- **Active corrosion sites (ACS):** Specific zones at the interface between the remaining metal and the corrosion layers stood out. They were identified as ACS similar to what was observed in a previous study of nails from the Bois-de-Châtel archaeological site [42]. These products (such as akageneite) tend to be very hygroscopic, and thus, important moisture changes were expected. The displacement maps excelled at highlighting the local changes on these sites.
- **Dense product layer (DPL):** Under the Limitos, the rest of the corrosion products in the dense product layer showed volume changes as well, especially in the case of BdC3 sample.
- **Transformed medium (TM):** The volume changes in the outer layer of corrosion, identified as the transformed medium layer, exhibited variability. The drying process generally led to contraction due to moisture loss. Conversely, the high RH treatment cause expansion in these products, attributed to moisture absorption.
- **Pores and cracks:** The analysis revealed an increase in voids and the emergence of new micro-cracks in the dried sample, suggesting that dehydration stresses contributed to material degradation and that pores filled with water were emptied. The sample exposed to high RH showed a complex moisture movement within the voids, likely due to the swelling of corrosion products chasing water from some voids and filling others.

As mentioned in the two sections above, it is currently difficult to accurately label the layer where the changes (loss/gain or expansion/shrinkage) occur. The next objective of the CORINT project is to identify various corrosion layers through the segmentation of the tomograms [42]. This would, in terms, allow us to precisely attribute the regions where the volume changes happen to a layer of the corrosion stratigraphy. Our segmentation results will be also confirmed with accompanying ex-situ chemical characterizations. In the meantime, the local displacement maps were interpreted by conservators and could already partially be attributed to specific layers.

**Fig. 10:**
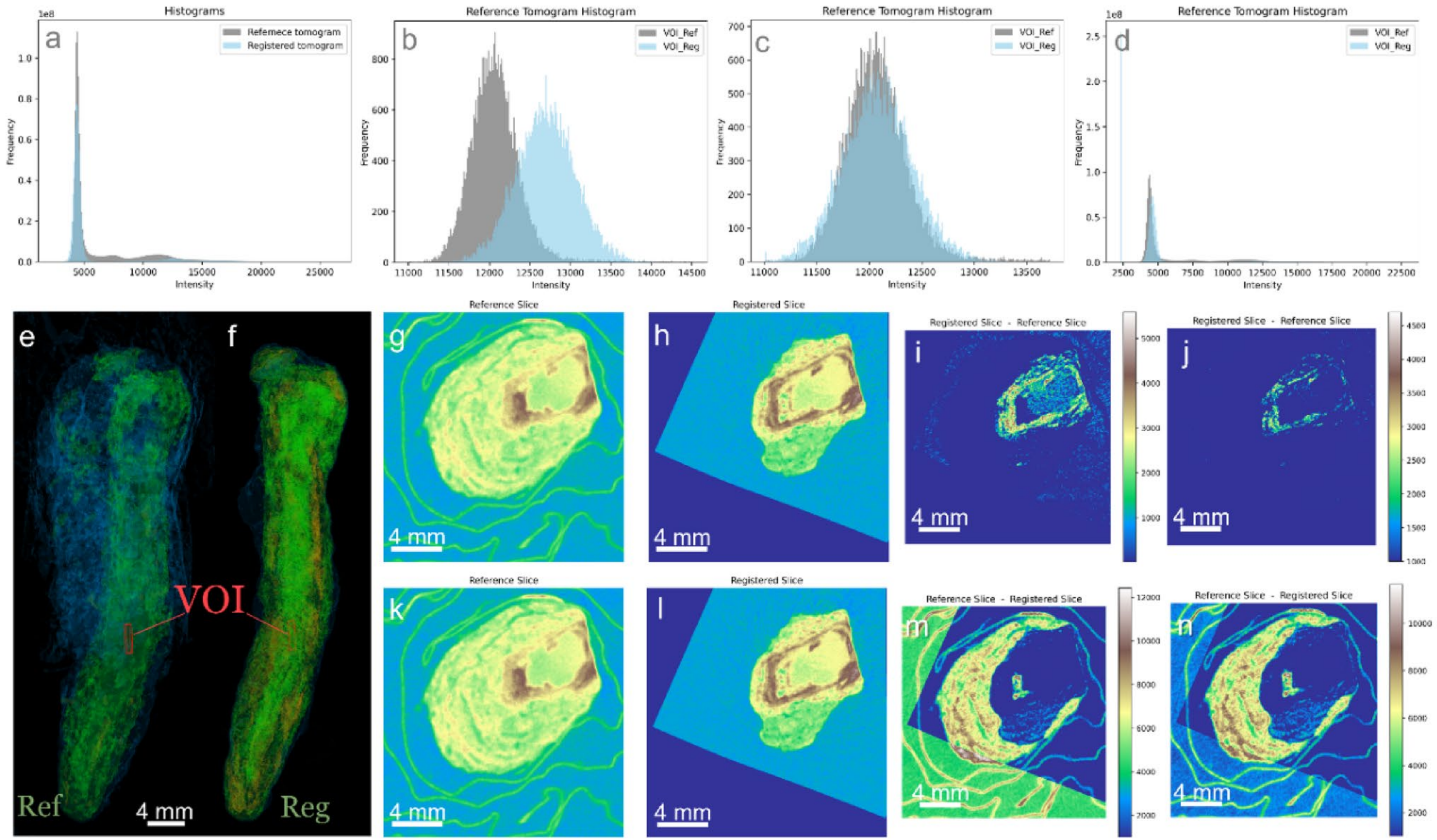
Illustrating the histogram matching process for the entire BdC4 sample (similar analyses to the one discussed in Fig. 4 of this article)

**Fig. 11:**
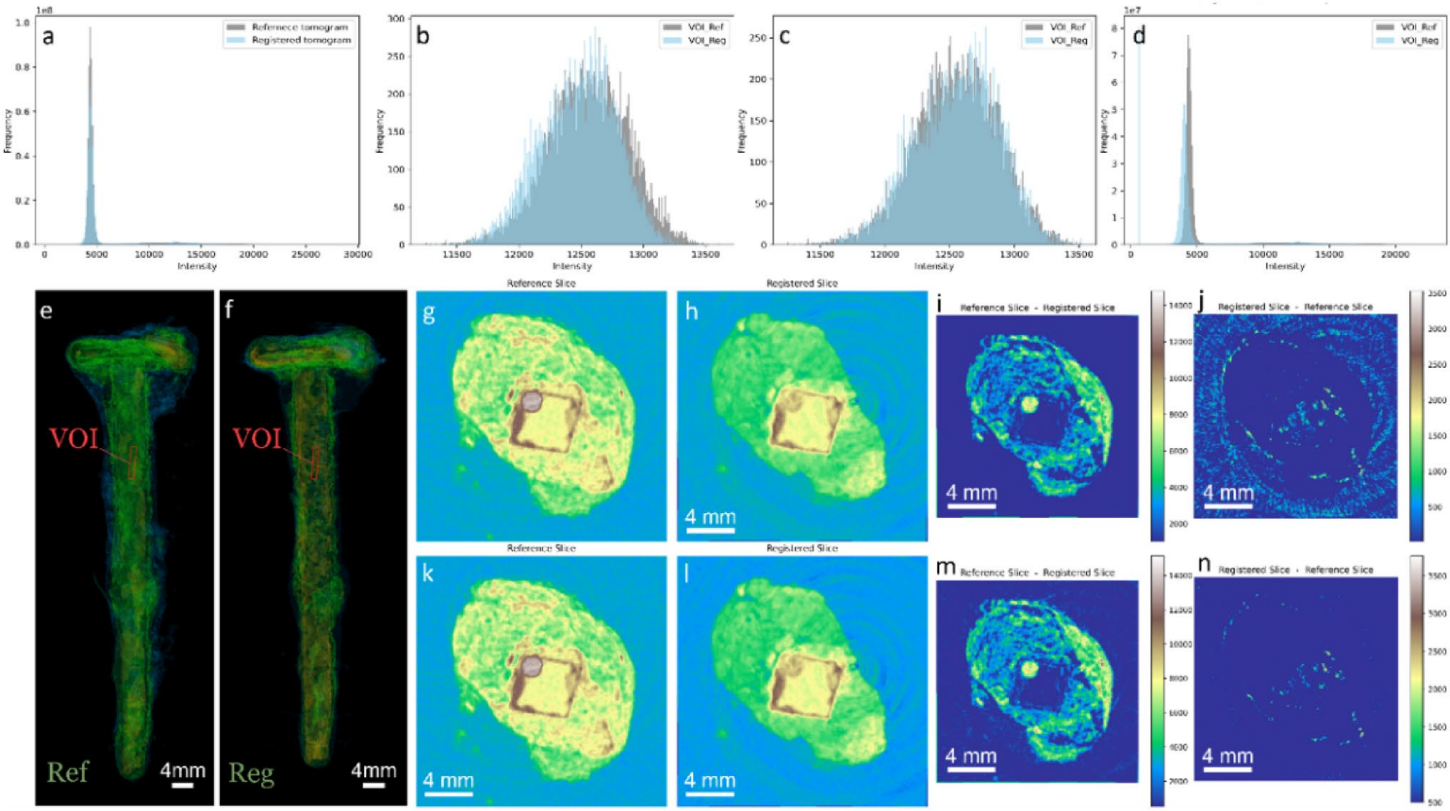
Similar plot as Fig. 10, but for sample BdC3

## Discussion

The application of image registration techniques that leveraged mutual information (MI) as the core metric was central to this study. This method proved to be instrumental in addressing the analytical complexities introduced by non-native materials (specifically, adhering soil clumps and wrapping material) that were initially present on the samples. Typically, such foreign substances could potentially obscure or distort the underlying features of interest, complicating the accurate assessment of the nail’s condition and the subsequent treatment effects. However, utilizing MI-based registrations marked a turning point in our methodological approach, enabling us to overcome these challenges effectively. By design, MI excels in scenarios where the direct comparison of intensity values across images is not viable due to variations introduced by external factors, such as foreign materials. Instead of relying on absolute intensity values, MI quantifies the statistical dependence between the intensity patterns of two images, making it exceptionally adapt at identifying correlations amidst noise and disturbance. This attribute of MI-based registration becomes particularly valuable when dealing with archaeological specimens that often come with unpredictable alterations and additions from their burial or storage. By accurately mapping regions that remain unchanged throughout the imaging and treatment processes, the MI metric ensures that the foundational structure of the archaeological artifacts is preserved in the analysis. This meticulous alignment allows for a precise comparison of pre-treatment and post-treatment states of the samples, providing a reliable basis for detecting even subtle changes within the materials. The ability to discriminate between unchanged and altered regions is crucial, as it directly influences the accuracy of subsequent analyses, such as the detection of local deformations and changes in material composition, resulting from the imposed environmental conditions. In our future publication we will compare various image registration metrics and various relevant parameters and their uncertainties, in more detail, for registering complex images from the same modality or dual modalities (X-ray and neutron tomograms).

The successful application of MI-based registrations underscores its utility in archaeological research, offering a reliable means of examining the subtle nuances of material degradation and preservation. Through this approach, we have gleaned valuable insights into the corrosion dynamics of Roman iron nails, setting a foundation for future conservation efforts grounded in a detailed understanding of how environmental changes and conservation treatments affect these ancient artifacts.

The two first experiments carried out for this paper specifically highlighted distinct response patterns. Exposure to high RH led to the local expansion in the corrosion products, linked with the absorption of moisture. This expansion could potentially fill some voids or cracks, highlighting the dynamic nature of corrosion processes under conditions of elevated moisture. The swelling of corrosion products under high RH conditions can exacerbate the corrosion process, potentially leading to further material degradation over time. Additionally, this volume uptake in the deeper layers of corrosion can cause further cracking and even loss of upper layers.

Conversely, drying treatments resulted in a decrease in the volume fraction of corrosion products, particularly those that are hygroscopic and tend to retain moisture. This decrease is attributed to the loss of bound water within these products, leading to a contraction of the corrosion layers and an increase in void spaces. The emergence of new micro-cracks or the enlargement of existing voids due to dehydration stresses further underscores the vulnerability of the corrosion matrix to drying conditions. In fact, such features may fragilize the artifact and introduce new pathways for oxygen and moisture penetration. This, in terms, would facilitate reactivation of corrosion processes under certain conditions. Indeed, though climate control in storage and exhibition spaces should be as stable as possible, fluctuations and even failures to maintain humidity low enough are to be expected. Hence, stabilization of the corrosion products, such as dechlorination and other treatments, is seen as a more reliable solution for a sustainable, long-term preservation of iron archaeological artifacts.

Recognizing the critical insights provided by this study, there is a clear opportunity to explore the effects of post-excavation changes and treatments across a broader range of archaeological samples. Future studies will aim to extend investigations on several similar samples to assess more comprehensively the impact of environmental conditions on the state of such artifacts.

By undertaking these future studies, we aim to build upon the foundational knowledge established in the current research, advancing the field of archaeological conservation. A more comprehensive assessment of the impact of environmental conditions on archaeological samples will equip conservators with the information needed to take proper decisions about the care and preservation of these invaluable artifacts.

## Conclusion

This study exemplified how neutron computed tomography combined with extensive image registration techniques can give insights into the corrosion dynamics in archaeological Roman iron nails under various environmental conditions. These two first experiments showed the effects of fast-drying and high relative humidity (RH) with a new, fully non-invasive 3D visualization and quantification of structural changes, corrosion site activation, and moisture variations. This new tool will have profound implications for the field of archaeological conservation, enabling further in-depth study of current best practices in terms of drying processes, desalination, and other conservation treatments. These findings will not only deepen our understanding of the material degradation processes but will also illuminate the pathways through which conservation strategies can be optimized to ensure the longevity and integrity of archaeological artifacts. These insights eventually pave the way for more nuanced and effective preservation methods. There are multiple takeaways from this preliminary study:

### Robustness of image registration

The utilization of mutual information (MI)-based affine registration proved crucial in overcoming the challenges of extraneous materials and sample alterations. This technique’s adaptability ensured high-quality alignment between tomograms, enabling accurate mapping of unchanged regions and effectively detecting localized changes due to treatments.

### Quantitative analysis of treatment effects

The study provided a comprehensive quantitative assessment of the changes within the nails’ corrosion products due to severe environmental changes. This analysis revealed the variability of the volume changes in the corrosion products and void spaces, underscoring the importance of moisture in corrosion dynamics. For example, it perfectly showed the moisture loss (BdC3, dried) or uptake (BdC4, exposed to high RH) within active corrosion sites (ACS) of the tested nails. Furthermore, we also quantified and linked the local physical changes as a result of these moisture dynamics.

Building on the foundation established by this research, future studies will aim to extend the observation across a broader range of archaeological samples and boundary conditions. By conducting similar investigations on multiple samples, we seek to assess the impact of environmental conditions more comprehensively on the preservation state of archaeological artifacts. We also aim at evaluating in detail the impact of various stabilization treatments such as desalination procedures. The quantitative assessment of local strains using neutron tomography is opening doors for reevaluating what is now conservation best-practice, finally giving a deep insight into the complex phenomena at play in archaeological iron corrosion post-excavation and post-treatment.

In the next stage of this research, advanced segmentation methods will be added to the existing protocol to precisely delineate corrosion layers, void spaces, and their changes in response to environmental boundary conditions. This final step will allow for a better quantitative assessment and contextualization of local intensity and physical variations within each phase (I.e., M, DPL, ACS, TM, S and pore space).

## Data Availability

The data is not available yet. This paper will be cited once the updated data will be published.

## Appendix A

See the figs. 10 and 11

## References

[1] C. Cooper, M. Milella, S. Lösch, The late iron age in Switzerland: a review of anthropological, funerary, and isotopic studies. Archaeol. Anthropol. Sci. **15**, 137 (2023). 10.1007/s12520-023-01838-w 37635748 10.1007/s12520-023-01838-w PMC10457247

[2] R.E. Hummel, The Iron Age, in *Understanding Materials Science: History · Properties · Applications*. ed. by R.E. Hummel (New York, NY, Springer, New York, 1998), pp.123–137

[3] L. Selwyn, C.C. Institute, Metals and Corrosion: A Handbook for the Conservation Professional, Canadian Conservation Institute, 2004.

[4] D.A. Scott, *Metallography and microstructure of ancient and historic metals* (Getty Conservation Institute in association with Archetype Books, Marina del Rey, CA, 1991)

[5] M. Rimmer, D. Thickett, D. Watkinson, H. Ganiaris, *Guidelines for the storage and display of archaeological metalwork* (English Heritage, London, 2013)

[6] W. Miller, R. Alexander, N. Chapman, I. McKinlely, J. Smellie, Chapter 3: Varieties of analogue studies, in *Geological Disposal of Radioactive Waste and Natural Analogues*. ed. by W. Miller, R. Alexander, N. Chapman, I. McKinlely, J. Smellie (Elsevier, 2000), pp.53–63

[7] D. Neff, P. Dillmann, L. Bellot-Gurlet, G. Beranger, Corrosion of iron archaeological artefacts in soil: characterisation of the corrosion system. Corrosion Sci. (2005). 10.1016/j.corsci.2004.05.029

[8] R. Bertholon, Characterisation and location of original surface of corroded metallic archaeological objects. Surf. Eng. **17**, 241–245 (2001). 10.1179/026708401101517863

[9] D. Watkinson, M.T. Lewis, Desiccated storage of chloride-contaminated archaeological iron objects. Stud. Conserv. **50**, 241–252 (2005). 10.1179/sic.2005.50.4.241

[10] L.S. Selwyn, P.J. Sirois, V. Argyropoulos, The corrosion of excavated archaeological iron with details on weeping and akaganéite. Stud. Conserv. **44**, 217–232 (1999). 10.1179/sic.1999.44.4.217

[11] D. Thickett. Post Excavation Changes and Preventive Conservation of Archaeological Iron, School of Biological and Chemical Sciences, Birkbeck College, University of London, London, 2012. LINK: https://production.english-heritage.org.uk/siteassets/home/learn/conservation/collections-advice--guidance/thickettthesisfinalversion.pdf

[12] D.E. Watkinson, M.B. Rimmer, N.J. Emmerson, The influence of relative humidity and intrinsic chloride on post-excavation corrosion rates of archaeological wrought iron. Stud. Conserv. **64**, 456–471 (2019). 10.1080/00393630.2018.1565006

[13] D. Watkinson, V. Neal. First aid for finds, 3rd ed ed., RESCUE--The British Archaeological Trust ; Archaeology Section of the United Kingdom Institute for Conservation with the Museum of London, Hertford, London, 1998.

[14] J.C. Thunberg, D.E. Watkinson, N.J. Emmerson, Desiccated microclimates for heritage metals: creation and management. Stud. Conserv. **66**, 127–153 (2021). 10.1080/00393630.2020.1799599

[15] A.B. Paterakis, L. Hickey-Friedman, Stabilization of Iron artifacts from kaman-kalehöyük: a comparison of chemical and environmental methods. Stud. Conserv. **56**, 179–190 (2011). 10.1179/204705811X13110713013236

[16] L. Selwyn, Overview of archaeological iron: The corrosion problem, key factors affecting treatment, and gaps in current knowledge, Metal 04: Proceedings of the International Conference on Metals Conservation, 2004. LINK: https://www.semanticscholar.org/paper/Overview-of-archaeological-iron%3A-the-corrosion-key-Selwyn/e9c074a939e5db6000354310f422da4d2077cda4

[17] D. Mannes, E. Lehmann, A. Masalles, K. Schmidt-Ott, K. Schaeppi, F. Schmid, S. Peetermans, K. Hunger, The study of cultural heritage relevant objects by means of neutron imaging techniques. Insight (2014). 10.1784/insi.2014.56.3.137

[18] D. Mannes, F. Schmid, J. Frey, K. Schmidt-Ott, E. Lehmann, Combined neutron and X-ray imaging for non-invasive investigations of cultural heritage objects. Phys. Procedia **69**, 653–660 (2015). 10.1016/j.phpro.2015.07.092

[19] D. Mannes, E.H. Lehmann, Neutron Imaging of Cultural Heritage Objects, in *Handbook of Cultural Heritage Analysis*. ed. by S. D’Amico, V. Venuti (Springer International Publishing, Cham, 2022), pp.211–237

[20] J.B.A. Maintz, M.A. Viergever, A survey of medical image registration. Med. Image Anal. **2**, 1–36 (1998). 10.1016/S1361-8415(01)80026-8 10638851 10.1016/s1361-8415(01)80026-8

[21] J.P.W. Pluim, J.B.A. Maintz, M.A. Viergever, Mutual-information-based registration of medical images: a survey. IEEE Trans. Med. Imaging **22**, 986–1004 (2003). 10.1109/TMI.2003.815867 12906253 10.1109/TMI.2003.815867

[22] P. Viola, W.M. Wells, Alignment by maximization of mutual information. Int. J. Comput. Vision **24**, 137–154 (1997). 10.1023/A:1007958904918

[23] F. Maes, A. Collignon, D. Vandermeulen, G. Marchal, P. Suetens, Multimodality image registration by maximization of mutual information. IEEE Trans. Med. Imaging **16**, 187–198 (1997). 10.1109/42.563664 9101328 10.1109/42.563664

[24] C. Studholme, D.L.G. Hill, D.J. Hawkes, An overlap invariant entropy measure of 3D medical image alignment. Pattern Recogn. **32**, 71–86 (1999). 10.1016/S0031-3203(98)00091-0

[25] T.C. Chu, W.F. Ranson, M.A. Sutton, Applications of digital-image-correlation techniques to experimental mechanics. Exp. Mech. **25**, 232–244 (1985). 10.1007/BF02325092

[26] A.W. Toga, P.M. Thompson, The role of image registration in brain mapping. Image Vis. Comput. **19**, 3–24 (2001). 10.1016/S0262-8856(00)00055-X 19890483 10.1016/S0262-8856(00)00055-X PMC2771890

[27] S. Klein, M. Staring, K. Murphy, M.A. Viergever, J.P.W. PluimElastix, A toolbox for intensity-based medical image registration. IEEE Trans. Med. Imaging **29**, 196–205 (2010). 10.1109/TMI.2009.2035616 19923044 10.1109/TMI.2009.2035616

[28] P. Blanc et al. Chronique des fouilles archéologiques 2017, Bulletin de l’Association Pro Aventico, 58, pp. 271–341. (2017) LINK: https://www.e-periodica.ch/digbib/view?pid=bpa-001%3A2017%3A58#4

[29] E.H. Lehmann, P. Vontobel, L. Wiezel, Properties of the radiography facility neutra at sinq and its potential for use as european reference facility. Nondestruct. Test. Eval. **16**, 191–202 (2001). 10.1080/10589750108953075

[30] C. Carminati, P. Boillat, F. Schmid, P. Vontobel, J. Hovind, M. Morgano, M. Raventos, M. Siegwart, D. Mannes, C. Gruenzweig, P. Trtik, E. Lehmann, M. Strobl, A. Kaestner, Implementation and assessment of the black body bias correction in quantitative neutron imaging. PLOS One (2019). 10.1371/journal.pone.0210300 10.1371/journal.pone.0210300 PMC6319815 30608985

[31] A.P. Kaestner, MuhRec—a new tomography reconstructor. Nucl. Instrum. Methods Phys. Res., Sect. A **651**, 156–160 (2011). 10.1016/j.nima.2011.01.129

[32] K. Marstal, F. Berendsen, M. Staring, S. Klein, SimpleElastix: A User-Friendly, Multi-lingual Library for Medical Image Registration, 2016 IEEE Conference on Computer Vision and Pattern Recognition Workshops (CVPRW), pp. 574–582. (2016). 10.1109/CVPRW.2016.78

[33] B. Lowekamp, D. Chen, L. Ibanez, D. Blezek, The design of simpleITK. Front. Neuroinform. (2013). 10.3389/fninf.2013.00045 10.3389/fninf.2013.00045 PMC3874546 24416015

[34] S. Klein, J.P. Pluim, M. Staring, M.A. Viergever, Adaptive stochastic gradient descent optimisation for image registration. In: International Journal of Computer Vision, **81**, pp. 227–239. (2009). 10.1007/s11263-008-0168-y.

[35] W.M. Wells, P. Viola, H. Atsumi, S. Nakajima, R. Kikinis, Multi-modal volume registration by maximization of mutual information. Med. Image Anal. **1**, 35–51 (1996). 10.1016/S1361-8415(01)80004-9 9873920 10.1016/s1361-8415(01)80004-9

[36] G. Zhang, Y. Chen, More informed random sample consensus, 2020 8th International Conference on Control, Mechatronics and Automation (ICCMA), IEEE, pp. 197–201. (2020). 10.48550/arXiv.2011.09116

[37] O. Zachariadis, A. Teatini, N.V. Satpute, J. G’omez-Luna, O. Mutlu, O.J. Elle, J. Olivares, Accelerating B-spline interpolation on GPUs: application to medical image registration. Comput. Methods Programs Biomed. (2020). 10.1016/j.cmpb.2020.105431 10.1016/j.cmpb.2020.105431 32283385

[38] B. Zitová, J. Flusser, Image registration methods: a survey. Image Vis. Comput. **21**, 977–1000 (2003). 10.1016/S0262-8856(03)00137-9

[39] A. Sotiras, C. Davatzikos, N. Paragios, Deformable medical image registration: a survey. IEEE Trans. Med. Imaging **32**, 1153–1190 (2013). 10.1109/TMI.2013.2265603 23739795 10.1109/TMI.2013.2265603 PMC3745275

[40] P. Burt, E. Adelson, The laplacian pyramid as a compact image code. IEEE Trans. Commun. **31**, 532–540 (1983). 10.1109/TCOM.1983.1095851

[41] D. Rueckert, L.I. Sonoda, C. Hayes, D.L.G. Hill, M.O. Leach, D.J. Hawkes, Nonrigid registration using free-form deformations: application to breast MR images. IEEE Trans. Med. Imaging **18**, 712–721 (1999). 10.1109/42.796284 10534053 10.1109/42.796284

[42] E. Granget, O. Cocen, M. Shakoorioskooie, Z. Qjanru, M.N. Lumongsod-Thompson, A. Kaestner, D. Mannes, L. Brambilla, Development of a Quantitative Multimodal Imaging Technique for In-situ Study of Iron Archaeological Artefacts, in: IMEKO (Ed.) IMEKO TC4 International Conference on Metrology for Archaeology and Cultural Heritage, Rome, Italy, 2023. 10.21014/tc4-ARC-2023.078

